# Broad‐Spectrum Antiviral Activity of the Orally Bioavailable Antiviral ATV014 Against Multiple Coronaviruses

**DOI:** 10.1002/mco2.70186

**Published:** 2025-04-15

**Authors:** Sidi Yang, Kun Li, Qifan Zhou, Xumu Zhang, Deyin Guo

**Affiliations:** ^1^ Guangzhou National Laboratory Guangzhou International Bio Island Guangzhou Guangdong China; ^2^ Shenzhen Key Laboratory of Small Molecule Drug Discovery and Synthesis Department of Chemistry College of Science, Academy for Advanced Interdisciplinary Studies and Medi‐X Pingshan Southern University of Science and Technology Shenzhen Guangdong China

1

Dear Editor,

Coronaviruses (CoVs) frequently cross species barriers, complicating the development of targeted antiviral treatments and making it challenging to control emerging viral infections. While most CoV strains tend to be specific to particular host species, zoonotic variants have the capacity to rapidly adapt to new hosts, often resulting in severe disease outbreaks. Over the past two decades, three new human CoVs have emerged: Severe Acute Respiratory Syndrome CoV (SARS‐CoV) in 2002, Middle East Respiratory Syndrome CoV (MERS‐CoV) in 2012, and SARS‐CoV‐2 in 2019, all of which caused significant health impacts in human populations. At present, no antiviral therapies have been approved that offer broad‐spectrum efficacy against multiple CoV strains in humans. The development of a therapeutic agent with wide‐ranging antiviral activity against CoVs would address a critical medical gap and prove invaluable in managing potential future outbreaks of novel CoV strains.

We recently reported the potent anti‐SARS‐CoV‐2 efficacy of ATV014 in preclinical studies [1]. ATV014 is a cyclohexane carboxylate prodrug of the parent compound 1ʹ‐CN‐4‐aza‐7,9‐dideazaadenosine C‐nucleoside (GS‐441524) (Figure 1A), which is currently undergoing Phase III clinical trials for the treatment of both mild and severe COVID‐19. Like Mindeudesivir and ATV006 (the same as Obeldesivir developed by Gilead), ATV014 is an oral analog of remdesivir (RDV), a broad‐spectrum antiviral initially developed for the Ebola virus and later repurposed for COVID‐19. All three compounds were developed as part of ongoing efforts to improve the pharmacokinetics, efficacy, and ease of administration of antiviral therapies for various viral infections, including those caused by CoVs [2, 3, 4]. Previous studies have shown that RDV exhibits broad‐spectrum antiviral activity against multiple RNA viruses, including CoVs, filoviruses, pneumoviruses, arenaviruses, and paramyxoviruses [5]. In light of this, we aimed to assess the antiviral potency and breadth of activity of ATV014 against several CoVs.

**FIGURE 1:**
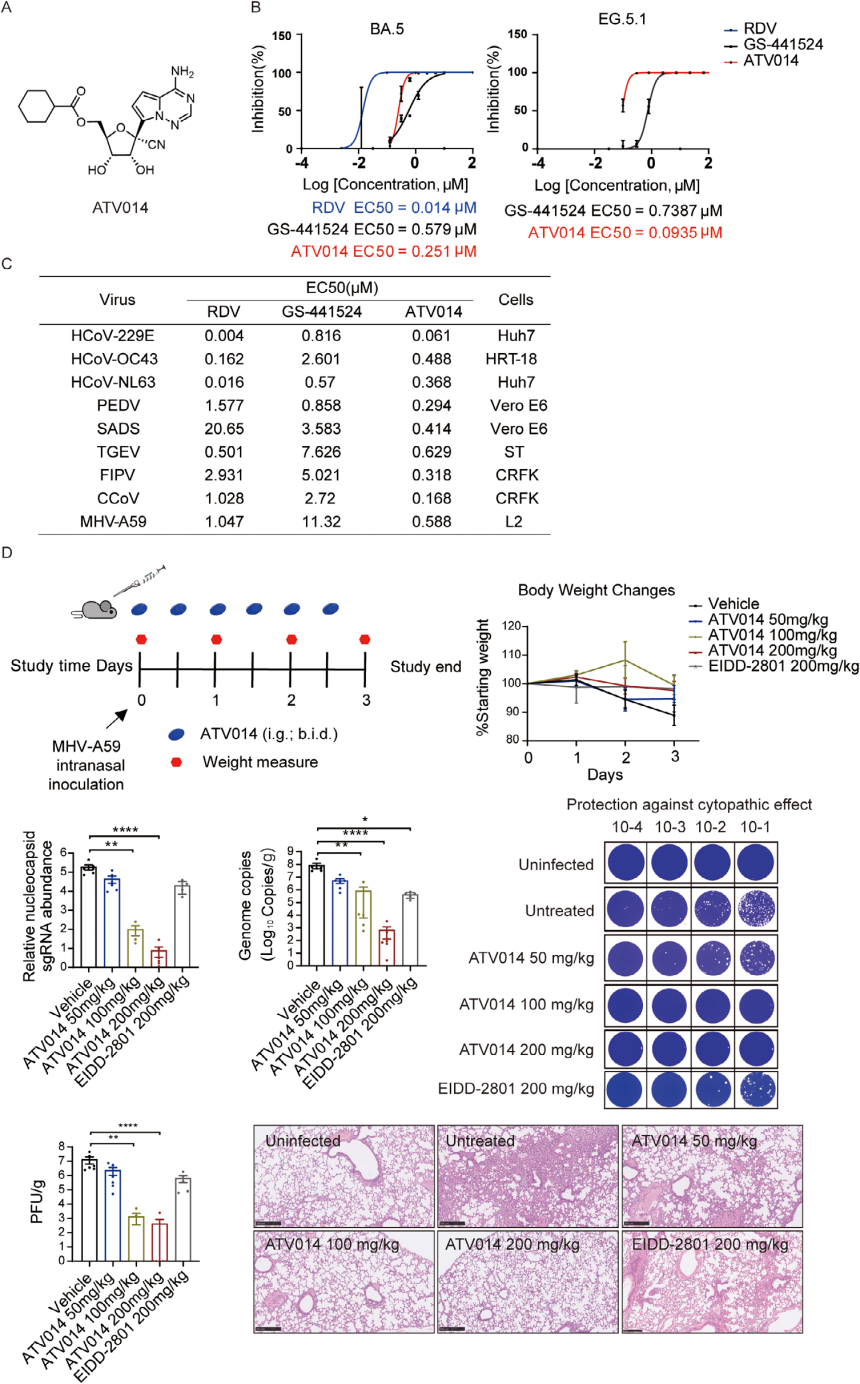
The oral prodrug ATV014 is an effective inhibitor of multiple coronaviruses. (A) The chemical structures of ATV014. (B, C) Antiviral activities of remdesivir (RDV), GS‐441524, and ATV014 against BA.5 and EG.5 (in A549‐hACE2 cell line) variants of SARS‐CoV‐2 (B), HCoV‐229E, HCoV‐OC43, and HCoV‐NL63, PEDV, SADS, and TGEV, FIPV, CCoV, and MHV‐A59 (C). (D) Experimental timeline for MHV‐A59 infection in C57BL/6J mice. Mice were intranasally inoculated with the MHV‐A59 (10^5^ PFU per mouse) and were treated with vehicle (control) or ATV014 (50, 100, or 200 mg/kg orally, BID) or EIDD‐2801 (200 mg/kg orally, BID) starting at the time of infection (*n* = 6 mice per group). Change in body weight was measured over time postinfection. MHV‐A59 genome copy numbers (left) and nucleocapsid (N) sgRNA were measured in the lungs at 3 dpi by qRT‐PCR. MHV‐A59 genome copy numbers were quantitated by qRT‐PCR with primer/probes targeting the N gene. The detection limit of qRT‐PCR was 0.5 copies/µL. At 3 dpi, infectious titer levels were analyzed by plaque assay. Photomicrographs of hematoxylin and eosin‐stained mouse lung sections from 3 dpi. Error bars indicate SEM. Statistical analysis was conducted using a Kruskal–Wallis test with Dunn's correction for multiple comparisons. *p ≤ 0.05; **p ≤ 0.005; ****p ≤ 0.0001.

Our previous studies demonstrated that ATV014 effectively inhibited the replication of SARS‐CoV‐2, including its various variants of concern (VOCs), such as Beta, Delta, and Omicron, with greater potency than RDV or GS‐441524 [1]. Given the evolving nature of SARS‐CoV‐2 variants, we initially assessed the antiviral efficacy of ATV014 in A549‐hACE2 cells infected with the latest variants, including Omicron BA.5 and EG.5. As depicted in Figure 1B, ATV014 showed notably enhanced antiviral activity against BA.5 (EC50 = 0.251 µM) and EG.5 (EC50 = 0.0935 µM) compared to GS‐441524, which aligns with the findings from our earlier research. CoVs are classified into several genogroups, such as alpha, beta, gamma, and delta, with human pathogenic CoVs primarily belonging to the alpha (HCoV‐229E and HCoV‐NL63) and beta (HCoV‐OC43, HCoV‐HKU1, SARS‐CoV, SARS‐CoV‐2, and MERS‐CoV) subgroups. The RNA‐dependent RNA polymerase (RdRp) enzyme, specifically the nsp12 protein, is highly conserved across CoVs, particularly within the same genogroup. To determine whether ATV014 exhibits antiviral activity against other human‐infecting CoVs, we infected different cell types with two alpha CoVs (HCoV‐229E and HCoV‐NL63) and a beta CoV (HCoV‐OC43). The results revealed that ATV014 demonstrated a low micromolar EC50 value against HCoV‐229E, HCoV‐NL63, and HCoV‐OC43 (Figure 1C).

The evolution and emergence of novel viruses are enabled by frequent cross‐species transmission. CoVs in humans and domestic animals are closely related, raising concerns about cross‐species transmission. Then, porcine epidemic diarrhea virus (PEDV), swine acute diarrhea syndrome coronavirus (SADS), transmissible gastroenteritis virus (TGEV), feline infectious peritonitis virus (FIPV), and canine enteric coronavirus (CCoV) were used to assess the inhibitory activity of ATV014 against different CoVs in domestic mammals. The compound showed improved potency against animal CoVs as compared to GS‐441524. Among them, ATV014 had an overall > 2.5‐fold, > 8‐fold, and > 12‐fold potency improvement in inhibiting the replication of PEDV, SADS, and TGEV, with EC50 values reaching 0.294, 0.414, and 0.629 µM, respectively. Meanwhile, ATV014 exhibited a low micromolar EC50 value with FIPV and CCoV, with EC50 values reaching 0.318 and 0.168 µM, respectively. Furthermore, the antiviral activities of ATV014 against mouse hepatitis virus (MHV‐A59) (EC50 = 0.588 µM) were improved compared to RDV and GS‐441524 (Figure 1C). Together, these data suggest that ATV014 can inhibit a broad range of CoVs, including circulating human and animal CoVs.

Next, we evaluated the in vivo therapeutic effectiveness of oral ATV014 using the animal CoV MHV‐A59 in C57BL/6J mice. The mice were intranasally infected with MHV‐A59 and subsequently treated with ATV014, vehicle, or EIDD‐2801, which served as a positive control (Figure 1D). While no significant differences in body weight changes were observed across the groups, the ATV014‐treated mice experienced less weight loss compared to the control group. To assess the extent of MHV‐A59 replication, we measured the levels of genomic RNA (gRNA), subgenomic RNA (sgRNA), and plaque‐forming units (PFU), with sgRNA serving as a marker for viral replication due to its discontinuous synthesis. At three days postinoculation (dpi), both gRNA and sgRNA levels were significantly lower in mice treated with ATV014 (100 and 200 mg/kg, BID). Moreover, virus titers in the majority of these mice were reduced to near undetectable levels, suggesting a substantial reduction in infectious virus in the lungs. Histopathological analysis revealed that ATV014 treatment protected lung tissue from interstitial inflammation and damage typically caused by MHV‐A59 infection (Figure 1D). Since MHV primarily induces hepatitis and liver damage in mice, we also assessed ATV014's antiviral effects on the liver. The results indicated that ATV014 effectively reduced both viral load and hepatic pathology (Figure S1). Collectively, these findings demonstrate that ATV014 exhibits strong antiviral efficacy against MHV in this mouse model.

Overall, we demonstrate that the nucleoside prodrug ATV014, currently in clinical development for the treatment of COVID‐19, can inhibit a broad range of CoVs, including both human and animal CoVs, across multiple in vitro systems, with sub‐micromolar EC50 values. ATV014 also showed efficacy against MHV‐A59 in a mouse model, confirming its broad‐spectrum anti‐CoV activity. The potency of orally bioavailable ATV014 against multiple CoVs underscores its potential as an effective antiviral treatment for both existing and emerging CoVs.

## Author Contributions

D.G. conceived and planned the overall structure of this project and edited the manuscript. S.Y. and K.L. carried out the experiments and drafted the manuscript. Q.Z. analyzed the data and synthesized the ATV014. X.Z. provided the experimental drug. All authors have read and approved the final manuscript.

## Ethics Statement

Approval of animal experiments was obtained from the Institutional Animal Welfare Committee of Guangzhou National Laboratory (#GZLAB‐AUCP‐2022‐10‐A06). All procedures used in animal studies complied with the guidelines and policies of the Animal Care and Use Committee of the respective research units. Work with the infectious MHV‐A59 strain under BSL2 conditions was approved by the Institutional Biosafety Committee (IBC) of Guangzhou National Laboratory.

## Conflicts of Interest

The authors declare no conflicts of interest.

## Supporting information



Supporting Information

## Data Availability

The data that support the findings of this study are available from the corresponding author upon request.
