# Supplementary material for: Broad‐Spectrum Antiviral Activity of the Orally Bioavailable Antiviral ATV014 Against Multiple Coronaviruses
Source: MedComm (2020). 2025 Apr 15;6(5):e70186. doi: 10.1002/mco2.70186 (PMC12000680; doi:10.1002/mco2.70186)
Supplement: Supplementary file 1 — Supporting Information [file MCO2-6-e70186-s001.docx]

**Supplementary Information for**

**Broad-spectrum antiviral activity of the orally bioavailable antiviral ATV014 against multiple coronaviruses**

Sidi Yang^1,#^, Kun Li^1, #^, Qifan Zhou^2^, Xumu Zhang^2^, Deyin Guo^1,*^

^1^ Guangzhou National Laboratory, Guangzhou International Bio Island, Guangzhou, Guangdong 510005, China

^2^ Shenzhen Key Laboratory of Small Molecule Drug Discovery and Synthesis, Department of Chemistry, College of Science, Academy for Advanced Interdisciplinary Studies and Medi-X Pingshan, Southern University of Science and Technology, Shenzhen, Guangdong 518000, China

^#^ These authors contributed equally

^*^ Correspondence to: Deyin Guo (guo_deyin@gzlab.ac.cn)

Materials and Methods

Cells lines and Viruses

A549-hACE2, Huh7, LLC-MK2, Vero E6, ST, CRFK, L2, Neuro-2a cells were maintained in Dulbecco’s Modified Eagle Medium (DMEM, CORNING) supplemented with 10% FBS (GIBCO) and 1% Penicillin-streptomycin (GIBCO). HRT-18 cells were maintained in RPMI 1640 (CORNING) supplemented with 10% FBS (GIBCO) and 1% Penicillin-streptomycin (GIBCO). All cell lines used in this study were purchased from ATCC and have undergone authentication by short tandem repeat (STR) analysis to confirm their identity and tested mycoplasma free. All cells were cultured at 37°C with 5% CO_2_. SARS-CoV-2 BA.5 and SARS-CoV-2 EG.5 strains were provided by the Center for Disease Control and Prevention of Guangdong Province. The HCoV-OC43, HCoV-229E and HCoV-NL63 strains were obtained from the Professor Jinchun Zhao (Guangzhou Medical University). MHV-A59 was originally provided by Professor Rong Ye (Wuhan University). CCoV, PEDV, TGEV, SADS-CoV and FIPV strains were obtained from the Guangdong Provincial Biotechnology Research Institute.

Antiviral activity assays in cell culture

Cells were pre-seeded to 96-well plates (5000 cells/100 μl/well) for 24 h and then infected at multiplicity of infection (MOI) of 0.01 with different coronavirus for 1 h at 37℃. Viral inoculum was removed, and cells were washed two times with PBS. Medium containing gradient concentration of compounds (ATV014, Remdesivir and GS-441524) or DMSO was added. For 2-5 days post infection, the cell supernatant was collected, antiviral activities were evaluated by quantification of viral copy numbers in the cell supernatant via real-time fluorescence quantitative PCR (qRT-PCR). The inhibition rate of nucleoside analogs was calculated based on the viral copy number, and the 50% effective concentration (EC50) was calculated with Graphpad Prism software 8.0. All experiments related to SARS-CoV-2 infections were performed at the Biosafety Level 3 (BSL-3) facilities and at least three independent experiments were performed.

qRT-PCR analysis

Total RNA from cell supernatant was extracted using Magbead Viral DNA/RNA Kit (CWBIO) following the manufacturer’s instructions. Total RNA from mouse tissue was extracted using [TRIzol](https://www.sciencedirect.com/topics/biochemistry-genetics-and-molecular-biology/trizol) (Invitrogen). For human coronavirus RNA quantification, total RNA was isolated from cell culture supernatant and the viral genomic RNA were determined by quantitative RT-PCR using primer pairs targeting viral nucleocapsid protein. The absolute quantification of human coronavirus RNAs were performed by HiScript® II U+ One Step qRT-PCR Probe Kit (Vazyme) using the corresponding probes. For MHV-A59 RNA quantification, total RNA was isolated from mouse tissue. The real-time PCR was performed using ChamQ Universal SYBR qPCR Master Mix (Vazyme). Primer sequences: SARS-COV-2-N (F-AAGAAATTCAACTCCAGGCAGC, R-GCTGGTTCAATCT GTCAAGCAG, Prb-TCACCGCCATTGCCAGCCA); HCoV-OC43 (F-TCGCTAGCAACCAGG CTGAT, R-TTGGGTCCCGATCGACAA, Prb-CAATACCCCGGCTGAC); HCoV-229E (F-TGGCACAGGACCCCATAAAG, R-CAACCCAGACGACACCTTCA, Prb-TGCAAAATTTAG AGAGCGTG); HCoV-NL63 (F-GTCACCTAGTTCTTCTGGTACTTCCA, R-GCTTATCAGCC CTGGGTTGA, Prb-AGAAACCTAATAAGCCTCTTT); MHV-A59-N (F-GGAACTTCTCGTT GGGCATTATACT, R-ACCACAAGATTATCATTTTCACAACATA, Prb-ACATGCTACGGCTC GTGTAACCGAACTGT); MHV-A59-sgN (F-TATAAGAGTGATTGGCGTCC, R-GAGTAA TGGGGAACCACACT); mGAPDH (F-AGAACATCATCCCTGCATCC, R-CACATTGGGGGT AGGAACAC); FIPV (F-AGCAACTACTGCCACRGGAT, R-GGAAGGT TCATCTCCCCAGT, Prb-AATGGCCACACAGGGACAACGC); CCoV (F-CAGTCTAGAAATAGATCTCAATC, R-GCTTGTTCTACACTGTCA, Prb-CCTTCTTGTTATTGGATTGTTGCCTTC); PEDV (F-CGCA AAGACT GAACCCACTAATTT, R-TTGCCTCTGTTGTTACTTGGAGAT, Prb-TGTTGCCAT TGCCACGACTCCTGC); TGEV (F-GCAGGTAAAGGTGATGTGACAA, R-ACATTCAGC CAGTTGTGGGTAA, Prb-TGGCACTGCTGGGATTGGCAACGA); SADS-CoV(F-CTGACTG TTGTTGAGGTTAC, R-TCTGCCAAAGCTTGTTTAAC, Prb-TCACAGTCTCGTTCTCGCAA TCA).

Mouse models of MHV-A59 infection

8-week-old wild-type C57BL/6J mice were purchased from SPF biotechnology Co. Ltd. (Beijing, China), were divided into five groups (n = 6 for each group), the vehicle group, the group receiving ATV014 (50 mg/kg, 100 mg/kg and 200 mg/kg orally, BID) or EIDD-2801 (200 mg/kg orally, BID). Mice were lightly anesthetized with isoflurane and infected intranasally with MHV-A59 (10^5^ PFU) in 40 μL DMEM. Two hours before MHV-A59 infection, mice were treated with vehicle, ATV014 and EIDD-2801 according to group description as described above. The weight of the mice was recorded daily. After 3 days post infection, mice were euthanized, lung and liver tissues were collected. Subsequently, the left lobe of lungs and the left lateral lobe of livers were fixed in 4% paraformaldehyde. The others lungs and livers were divided equally and then homogenized in PBS or TRIzol reagent for additional analyses. To observe the pathological changes of liver or lung tissue in mice, the mouse formalin-fixed and paraffin-embedded lung or liver tissues were sectioned to slices of about 4 μm and were stained with H&E. The images were scanned by NanoZoomer S360 (Hamamatsu Photonics). Approval of animal experiments was obtained from the Institutional Animal Welfare Committee of Guangzhou National Laboratory (approval number: GZLAB-AUCP-2022-10-A06). All procedures used in animal studies complied with the guidelines and policies of the Animal Care and Use Committee of the respective research units.

MHV-A59 plaque assay

L2 cells were grown in 12-well plates to 90% confluence and infected with 1 mL of media containing viruses at dilutions ranging from 10^1^ to 10^6^. After cultured 2 h at 37℃, the inoculate were removed and 1 ml of 1.3% methyl cellulose in DMEM with 2% FBS was overlaid onto cells for 2 days. For plaque staining, 1 mL of 0.5% crystal violet was overlaid onto cells. Six to eight hours later, the stained plaques were counted.

Statistical analysis

**
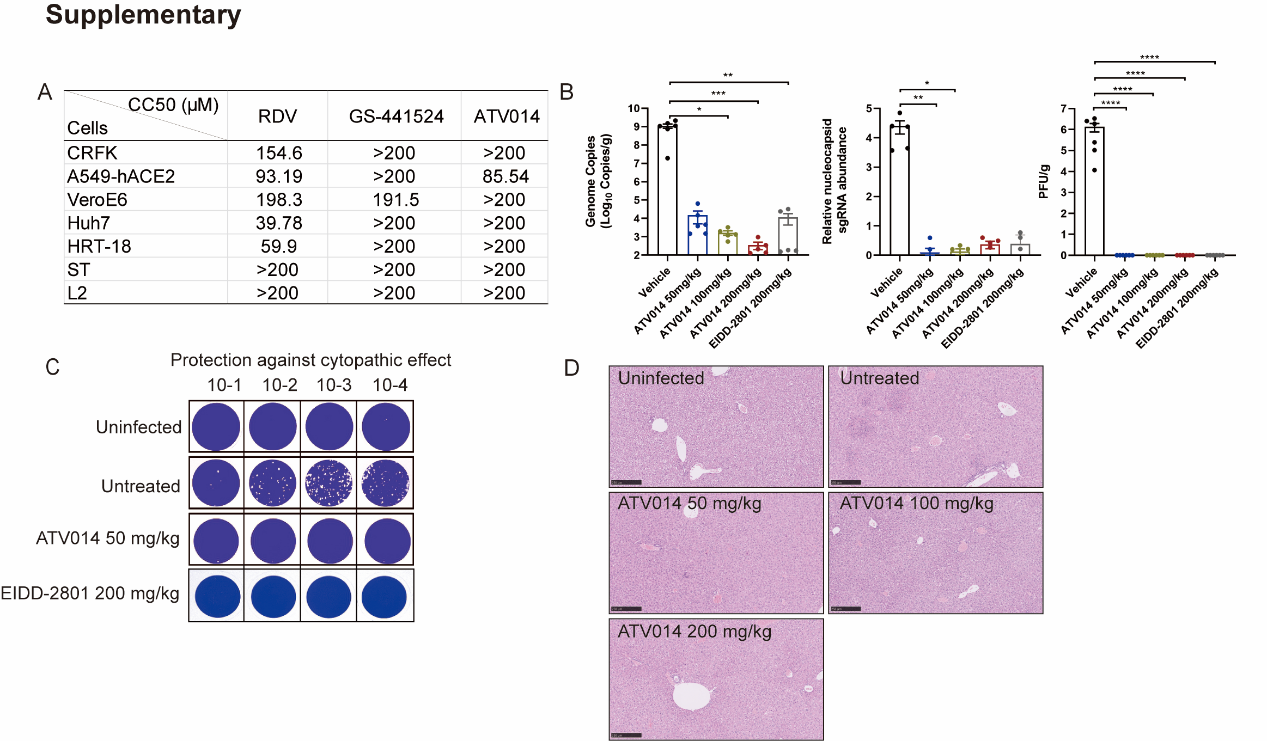
**All values are presented as means ± SEM of individual samples. Data analysis was performed with GraphPad Prism Software (GraphPad Software Inc., version 8.0). All experiments were performed at least in triplicate. Statistical significance for samples was identified using the Kruskal-Wallis test with Dunn's correction for multiple comparisons. *P* < 0.05 were considered statistically significant.

**Fig S1. Cell viability and effect of ATV014 on liver. (A)** Cell viability on different cell types**.** Mice were intranasally inoculated with the MHV-A59 (10^5^ PFU per mouse) and were treated with vehicle (control), ATV014 (50 mg/kg, 100 mg/kg or 200 mg/kg orally, BID) or EIDD-2801 (200 mg/kg orally, BID) starting at the time of infection (n=6 mice per group). **(B)** MHV-A59 genome copy numbers and nucleocapsid (N) sgRNA were measured in the livers at 3 dpi by qRT-PCR. MHV-A59 genome copy numbers were quantitated by qRT-PCR with primer/probes targeting the N gene. The detection limit of qRT-PCR was 0.5 copies/μL. **(C)** At 3 dpi, infectious titer levels was analyzed by plaque assay. **(D)** Photomicrographs of hematoxylin and eosin-stained mouse livers sections from 3 dpi. Error bars indicate SEM. Statistical analysis was conducted using a Kruskal-Wallis test with Dunn's correction for multiple comparison. **p*≤0.05; ***p*≤ 0.005; ****p*≤0.001, *****p*≤0.0001.
